# Fighting *Salmonella* Infantis: bacteriophage-driven cleaning and disinfection strategies for broiler farms

**DOI:** 10.3389/fmicb.2024.1401479

**Published:** 2024-05-15

**Authors:** Sandra Sevilla-Navarro, Jan Torres-Boncompte, Josep Garcia-Llorens, Mireia Bernabéu-Gimeno, Pilar Domingo-Calap, Pablo Catalá-Gregori

**Affiliations:** ^1^Centro de Calidad Avícola y Alimentación Animal de la Comunidad Valenciana (CECAV), Castellón, Spain; ^2^Instituto de Biología Integrativa de Sistemas, Universitat de València-CSIC, Paterna, Spain

**Keywords:** bacteriophage, autophages, *Salmonella*, multidrug resistance, biocontrol

## Abstract

**Introduction:**

*Salmonella* is a bacterium that can cause food-borne infections and is responsible for the most common gastrointestinal illnesses. The emergence of multi-drug resistant (MDR) strains worldwide is a major threat, representing a major challenge in public health. To reduce its incidence, the One Health approach is required, and the development of new biocontrol protocols will help prevent or eliminate the spread of *Salmonella*. Prevention measures, such as on-farm cleaning and disinfection protocols, are a crucial step in reducing infection to new flocks and eliminating bacteria that remain in the facilities. However, MDR *Salmonella* species, such as *S*. Infantis, are highly resistant to conventional cleaning and disinfection protocols, with an increased ability to persist in the broiler farm environment. The need for alternative biocontrol methods has led to the use of bacteriophages or phages, viruses that target bacteria, as promising tools. Thus, the aim of this study was to evaluate the efficacy of phages as a biocide against *S*. Infantis isolates in combination with cleaning and disinfection protocols in 10 commercial poultry farms.

**Methods:**

All commercial farms selected in this study had persistent *Salmonella*, even after the routinely used cleaning and disinfection procedures. In addition, *Salmonella* isolated before treatment were phenotypically characterized by antimicrobial resistance patterns.

**Results:**

The results showed that 100% of *S*. Infantis were resistant to at least one antibiotic, and > 70% were MDR. Phages were then isolated against the in-farm bacteria, purified, and multiplied for each poultry farm. The cleaning and disinfection protocols included the application of the lytic phages (vB_Si_CECAV_FGS009; vB_Si_CECAV_FGS017; vB_Si_CECAV_FGS029 and vB_Si_CECAV _FGS030) twice at 24-h intervals between cleaning and disinfection. Following the cleaning and disinfection procedures, *Salmonella* detection was reduced from 100% after cleaning to 36% after applying the phages and dropped to 0% after the final step of disinfection, thus eliminating *Salmonella* from the farm facilities.

**Discussion:**

This study demonstrates that bacteriophage application after cleaning and before disinfection enhances the removal of MDR *Salmonella* Infantis in commercial broiler farms, suggesting their use as biocontrol agents to reduce *Salmonella*, a major public health concern.

## 1 Introduction

*Salmonella* is the main pathogen involved in food-borne outbreaks worldwide. Although most cases of salmonellosis are mild, the severity of the disease depends on host factors and the *Salmonella* serovar, being life-threatening in some patients. Salmonellosis in humans is usually contracted through the consumption of contaminated food of animal origin (mainly eggs, meat, poultry, and milk) (World Health Organization, 2018). Control of food-borne *Salmonella* infections requires preventive measures involving all stages of the food chain, from agricultural production to food processing, manufacturing, and preparation, which demands effective food hygiene and water sanitation systems. Thus, *Salmonella* is a clear example where a “One Health” approach is essential, including new biocontrol tools to promote integrated strategies to reduce *Salmonella* in animals and limit transmission in terms of outbreak prevention (Silva et al., 2014).

There are more than 2,500 *Salmonella* serovars, with *Salmonella* Infantis being the third most frequently reported serovar in broilers, playing an important role in humans and animal health (García-Soto et al., 2020; EFSA and ECDC, 2021). The latest data from the European Food Safety Authority (EFSA) showed that 95.6% of the *Salmonella* isolates in broiler flocks were *S*. Infantis, being the most reported serovar, which implies an increase of 2.5% compared to the previous year (EFSA and ECDC, 2023). As for human cases, *S*. Infantis frequently causes food-borne illness, being the fourth most reported serovar in EU since 2011 (Newton et al., 2020; EFSA and ECDC, 2023). Although, *S*. Infantis has been isolated from swine and cattle, chicken products are the main vehicle for transmission of this serovar through the food supply chain to humans (Gu et al., 2020; EFSA and ECDC, 2023). The emergence of multi-drug resistant (MDR) strains is a global public health concern, as these are bacteria with a growing prevalence and increasing mortality and morbidity rates worldwide. Antimicrobial resistance (AMR) in food-borne pathogenic bacteria, such as *Salmonella,* is becoming one of the most concerning public health issues (Newton et al., 2020). The global problem of AMR is driving the search for novel treatments to control pathogenic MDR bacteria (Thanki et al., 2021). In fact, AMR is associated with more than 1.2 million deaths annually (Murray et al., 2022), and this number is estimated to increase to 10 million deaths annually by 2050 if effective solutions are not found and applied (O’Neill, 2014, 2016; Murray et al., 2022).

Since 2007, the strict measures carried out by the National *Salmonella* Control Programs (NSCP) according with the Regulation (EC) N. ° 2160/2003 (PNCS, 2020) have controlled the bacterium reaching the prevalence level indicated by the EU. However, a major problem arises due to the high persistence of MDR serovars such as *S*. Infantis in broiler chicken, which is nowadays one of the main threats to food health (Vandeplas et al., 2010; EFSA and ECDC, 2021). The persistence and resistance of *Salmonella* is commonly associated with the ability to form biofilms, surface-associated communities in an exopolysaccharide matrix formed by the bacteria (Steenackers et al., 2012). The complexity of the biofilm and the difficulties of conventional treatments to remove these structures require alternative tools to effectively remove biofilms.

Cleaning and disinfection (C&D) are considered a crucial step to reduce infection to new flocks and prevent the bacteria from remaining in the facilities (Corcoran et al., 2014). However, *S*. Infantis, due to its high resistance to disinfection, has been described as persistent in the environment, with an increase ability to persist on broiler farms even after conventional C&D protocols in biofilms (White et al., 2006; Corcoran et al., 2014; García-Soto et al., 2020). The persistence of *Salmonella* in poultry farms after the C&D could be related to inadequate procedures or due to the development of resistance to disinfectants (Davis et al., 2002; Gradel et al., 2005; Alba et al., 2020). Different disinfectants, such as glutaraldehyde, quaternary ammonium compounds, and formaldehyde have been used at the field level, with formaldehyde being one of the most effective antibacterials available (Drauch et al., 2020). However, concern about the danger to humans has led to a ban on its use on farms, motivating research into alternative methods, such as the use of phages (Gutiérrez et al., 2016; Sevilla-Navarro et al., 2020a). In this regard, a major challenge in eliminating bacterial biofilms is the need to remove the matrix and persistent cells. Common areas where *Salmonella* biofilms form in broiler farms include water systems (pipes, drinkers, tanks), feed lines and feeders, flooring and litter, and equipment and infrastructure (Maes et al., 2019; Pang et al., 2023). These areas can harbor biofilms due to contamination with *Salmonella*, posing risks for both horizontal and vertical transmission. Thus, bacteriophages or phages, viruses that target bacteria, show interesting features in biofilm removal (Szafrański et al., 2017; Ferriol-González and Domingo-Calap, 2020). Their ability to produce specific phage-derived enzymes that allow them to actively penetrate and disrupt biofilms, in addition to their multiplicity at the infection site and their high specificity, makes phages a very promising biocontrol tool. Phages are found in all environments in which bacteria grow and replicate, co-evolving together and contributing to limiting their over-spreading and maintaining the equilibrium in ecosystems (D’accolti et al., 2021).

Biocontrol phage-based strategies are not new. In countries such as the United States (US), the Food and Drug Administration (FDA) approved the use of a phage cocktail encompassing six phages (ListShield^TM^) as a direct food additive for the control of *Listeria monocytogenes* in poultry and ready-to-eat meat products; and the use of the phage cocktail SALMONELEX^TM^ against *Salmonella* (Micreos Food Safety, Wageningen, The Netherlands) for food processing control on beef and vegetables (Federal Register, 2006). However, phages are not yet authorized in EU by the EFSA nor EMA (European Medicine Agency) although some phage-based products are made in Europe. One of the advantages of phages is their host specificity. However, this could difficult the efficacy of broad-spectrum cocktails to not be effective enough against all variants of a given bacteria (Abedon et al., 2021). An alternative is the use of autophages (APs), phages isolated from the same environment where the target bacterium has been isolated (Sevilla-Navarro et al., 2018). This can prevent and reduce *Salmonella* from farms, contributing to reduce its overall incidence. In this context, and in the same way that phages are used as food biocontrol, their use is being encouraged as a complementary tool to the cleaning and disinfection of farm facilities (Sevilla-Navarro et al., 2020b; D’accolti et al., 2021). The use of phage products helps to reduce persistent bacteria through the active elimination of biofilms. However, the fact that each farm has its own idiosyncrasies coupled with the fact that phages are extremely specific toward their host, makes it necessary to produce a farm-specific and cost-effective product capable of controlling the presence of bacteria, such as the use AP (Sevilla-Navarro et al., 2018). A combination of interventions is needed to prevent further spread and persistence of *S*. Infantis in broiler flocks (Newton et al., 2020). However, very little has been described on the efficacy of APs as field-level sanitizers in poultry farms.

Therefore, the aim of this study was to evaluate the efficacy of phage biocontrol against *S*. Infantis isolates in combination with cleaning and disinfection protocols in commercial poultry farms. The results are encouraging and support the use of APs in the control of persistent and resistant *S*. Infantis in the field.

## 2 Materials and methods

### 2.1 Selection of the broiler houses

During this study, 10 broiler houses which housed a total of 22 flocks were selected and sampled from 2020 to 2021. Poultry farms used in this study were in the North and East of Spain. The procedures carried out in this study were approved by the Ministry of Agriculture of each of the respective communities (based on the CSV: 2AD5M8FG-DALESJC1-7DA55S99). All houses included in this study had persistent *Salmonella* bacteria even though after their conventional C&D procedure. Furthermore, at the end of each trial, phages were inactivated from farm facilities after application of disinfectants.

### 2.2 *Salmonella* isolation

All samples were analyzed according to the ISO 6579:2017 (Mooijman et al., 2019) and serotyped following the Kauffman-White-Le Minor technique (Grimont and Weill, 2007). Swab cloths and boot swab samples were diluted 1:10 (w/v) in BPW (Buffered Peptone Water ISO, VWR Chemicals, Barcelona, Spain). Samples were then incubated at 37°C for 18 ± 2 h. The pre-enriched samples were transferred into Modified Semi-Solid Rappaport Vassiliadis agar plate (MSRV, Difco, Valencia, Spain), which was incubated at 41.5 ± 1°C for 24–48 h. Suspicious plates obtained in MSRV were transferred into Xylose–Lysine– Deoxycholate (XLD, Liofilchem, Valencia, Spain), and ASAP (bioMerieux, Madrid, Spain), and then incubated at 37 ± 1°C for 24 h. After the incubation, 5 typical colonies of *Salmonella* were selected and streaked into nutrient agar plates (Scharlab, Barcelona, Spain) at 37 ± 1°C for 24 ± 3 h (Mooijman et al., 2019). *Salmonella* strains isolated were serotyped according to the Kauffman-White- Le Minor technique (Grimont and Weill, 2007).

### 2.3 Antimicrobial susceptibility test of the isolated bacteria

*Salmonella* target phage strains were characterized according to the ISO 20776–1:2006, by a commercially available microtitre plates Sensititre™ EUVSEC (Thermo Scientific, East Grinstead, United Kingdom). To this end, *Salmonella* Sensititre Plates (Gram Negative MIC Plate) were used to assess antimicrobial susceptibility of isolated strains. The antibiotics selected were those from the antimicrobial panel determined by the Commission Implementing Decision of November 2020 on the monitoring and reporting of antimicrobial resistance in zoonotic and commensal bacteria (Commission Implementing Decision, 2020). This panel, included, Ciprofloxacin (CIP, 0.015–8 μg/mL) and Nalidixic Acid (NAL, 4–128 μg/mL); 2 B-lactams: Meropenem (MERO, 0.03–16 μg/mL) and Ampicillin (AMP, 1–64 μg/mL), one phenicol: Chloramphenicol (C, 8–128 μg/mL); one pyrimidine: Trimethoprim (TM, μg/mL); one tetracycline: Tetracycline (TET, μg/mL); one macrolide: Azithromycin (AZM, 2–64 μg/mL); one glycylcycline: Tigecycline (TGC, 0.25–8 μg/mL); 2 cephalosporin: Ceftazidime (CAZ, 0.5–8 μg/mL) and Cefotaxime (CTX, 0.25–4 μg/mL); one polymyxin: Colistin (COL, 1–16 μg/mL); one potentiated sulfonamide: Sulfamethoxazole (SMX, 8–1,024 μg/mL), and one aminoglycoside: Gentamicin (GN, 0.5–32 μg/mL). Epidemiological cutoff values (ECOFF) were taken to determine resistance against the antibiotics analyzed. These values were established by the European Committee on Antimicrobial Susceptibility Testing (EUCAST) and recommended by UE Commission Decision 179/2020 (Commission Implementing Decision, 2020). The values not included in this legislation (Azithromycin and Sulfamethoxazole) were assessed following National Committee for clinical Laboratory Standards (NCCLS) criteria (Clinical and Laboratory Standards Institute, 2024). MDR was defined as acquired resistance to at least one agent in 3 or more antimicrobial classes (ECDC, 2016).

### 2.4 Isolation and phenotypic characterization of *Salmonella* Infantis phages

Isolation and purification of phages was done using the bacterial host of each selected farm according to Sevilla-Navarro et al. (2020a,b). Phages were isolated from feces collected from the selected farms by an enrichment procedure, with the aim of applying an AP (Sevilla-Navarro et al., 2018). To do so, 10 g of each feces sample from each farm were diluted in 90 mL of Luria Bertani (LB) (VWR Chemicals, Barcelona, Spain) and incubated along with each specific *Salmonella* serovar overnight at 37°C. After incubation, 2 mL of this enrichment culture was centrifuged 16,000 × g for 5 min. The supernatant was then filtered through a 0.22 μm membrane. Phages were isolated and purified in a spot test by the double agar method. Briefly, bacterial suspensions of each serovar were adjusted to an optical density (OD) 600 nm of 0.2 nm (~108 CFU/mL) in LB and incubated at 37°C for 4 h. Then, 200 μL of cultures were added to 5 mL of LB agar (LB with 0.6% agar) tempered to 45°C and poured onto previously prepared and dried LB basal agar (with 1.6% agar). Then, 10 μL of each filtrate was spotted onto the surfaces of *Salmonella* lawns and incubated overnight at 37°C. After the incubation, morphologically different plaques were selected and resuspended in 50 μL of PBS. Ten-fold serial dilutions of the phage suspension were plated by the double agar layer method, and phages that produced clear plaques were selected.

### 2.5 Genomic analysis of *Salmonella* Infantis phages

To extract DNA, 10 μL was used of highly concentrated lysates for DNAse I treatment to remove bacterial host. Subsequently, capsids were enzymatically digested using Proteinase K, and the resulting phage DNA was purified using the commercial DNA Clean & Concentrator-5 kit (Zymo Research, USA). Library was prepared with the Nextera XT Library prep kit and used for sequencing using Illumina MiSeq technology (250 bp paired-end reads). Quality control of raw data was done with fastp and resulting fastq files were used to generate a *de novo* assembly with SPAdes version: 3.13.1 (only-assembler mode) (Bankevich et al., 2012). Contigs were analyzed to discard those shorter than 1 Kbp and coming from bacterial host leading to one contig with high length and k-mer coverage that was taken as the phage genome. A BLAST comparison against the nucleotide database was then performed to determine the closest relative of each genome and assign a preliminary taxonomic classification to each phage (Altschul et al., 1990). The beginning of related representative phages was used to reorder the genomes. Afterwards, the rearranged genomes were corrected with Pilon v1.24 and coverage was evaluated by mapping the trimmed reads with BBMap.sh v38.95 (Bushnell, 2014; Walker et al., 2014). For structural and functional annotation, Pharokka v1.4.1 was used with default parameters and each sequenced coding was associated with a Phrog functional group (Bouras et al., 2023). Phage depolymerases were predicted using the machine learning tool DePP (web version 1.0.0) and protein products with at least a 90% of probability of having depolymerase activity were considered potential depolymerases (Magill and Skvortsov, 2023). Phage safety was assessed using Pharokka in combination with PhageLeads, to determine the presence of temperate markers and antimicrobial resistance and virulence genes (Yukgehnaish et al., 2022). Additionally, phage lifestyle was predicted with PhaTYP (web version from Phage BOX) (Shang et al., 2023). Finally, isolated phage genomes were compared by BLAST and represented with gggenomes v1.0.0 (Hackl et al., 2023). To confirm phage taxonomy, two different approaches were used using Refseq sequences from phages belonging to the preliminary assigned taxonomic rank. Firstly, a single-gene phylogeny using the large subunit of terminase protein was performed using ClustalW v2.1 as the aligner and IQTREE v1.6.12 for phylogenomic inference with 1,000 ultrafast bootstrap replicates (Thompson et al., 1994; Trifinopoulos et al., 2016). The maximum likelihood tree was constructed using ITOL v5 fixing the midpoint as the root and removing branches with a value of bootstrap lower than 90 (Letunic and Bork, 2021). Secondly, VIRIDIC v1.1 was used for calculating viral intergenomic similarity and clustering in species and genera (95 and 70% thresholds respectively) (Moraru et al., 2020). Genus’s clustering was represented with a circle packing using RawGraphs v2.0 (Mauri et al., 2017).

### 2.6 Transmission electron microscopy of the isolated phages

Phages were visualized by transmission electron microscopy (TEM) (FEI Tecnai G2 Spirit Biotwin, 120 KW) in the Centro de Investigación Principe Felipe (CIPF, Valencia, Spain). To this end, 10 μL from the AP with a concentration of 10^8-9^PFU/mL was fixed in an aqueous solution of paraformaldehyde (2%). A 7.2 V glow was discharged on samples placed on the MESH Cooper grid and incubated in the grids for 15 min. Then, samples were washed in phosphate buffer 0.1 M for 2 min and fixed with glutaraldehyde (1%). Samples were negatively stained with uracil acetate (1%) and incubated with methyl cellulose (1%) for 30 s. Samples were dried until use (Li et al., 2016; Sevilla-Navarro et al., 2018).

### 2.7 Determination of the phage host range (HR) and efficiency of plating (EOP)

A total of 41 *Salmonella* isolates were tested, with a focus on *S*. Infantis strains from different years (2013, *n* = 9; 2018, *n* = 10; and 2023, *n* = 10). Additionally, various *Salmonella* serovars with likely antigenic formulae isolated in 2023 were selected, including *S*. Ohio, *S*. Mikawasima, *S*. Mbandaka, and *S*. Newport. The assess the bacterial susceptibility to phages, a spot titration protocol was used that allow us to determine both host range and relative EOP (Gibson et al., 2019). To this end, 10 μL of serial 10-fold dilutions of each phage were spotted on freshly seeded *Salmonella* spp. lawns and incubated overnight at 37°C. After incubation, the host range and titer were determined by formation of individual plaques within the area of the spot at terminal dilution. EOP was calculated by dividing the titer of the phage at the terminal dilution on the challenge strain by the titer of the same phage on its host strain. Phages were classified as “highly virulent” for ratio > 0.5, “medium virulent” for ratios ≥ 0.1 and ≤ 0.5, “avirulent but active” for ratios >0.001 and < 0.1 and “inefficient” for ratios ≤0.001 or no plaques detected.

### 2.8 Phage multiplication

A total of 500 mL of each bacteriophage (AP) was multiplied in its specific host to produce high-titer lysates (10^^9–10^ PFU/mL). For this, 500 mL of LB medium supplemented with 5 mL of each host culture was grown to mid-log phase (OD600 = 0.2) at 37°C. Subsequently, each phage lysate was added at a multiplicity of infection (MOI) of 0.1. The bacterium-phage mixture was then incubated under agitation (180 rpm) for up to 4 h (vB_Si_CECAV_FGS009, vB_Si_CECAV_FGS017) and up to 6 h (vB_Si_CECAV_FGS029, and vB_Si_CECAV_FGS030), until complete lysis of the bacterial culture was observed. Following incubation, lysates were centrifuged at 10,000 × g for 5 min and filtered using 0.2 μm filters (Nalagene, ThermoFisher) to remove any remaining bacterial cells and debris. Subsequently, 10 μL of each fold dilution were spotted onto double agar layers and incubated overnight at 37°C for quantification of phage concentration via plaque assays and to confirm the absence of viable *Salmonella* cells. Phages were stored at refrigeration temperatures (4°C) until they were used.

### 2.9 Cleaning and disinfection procedure with AP in field conditions

**Figure 1:**
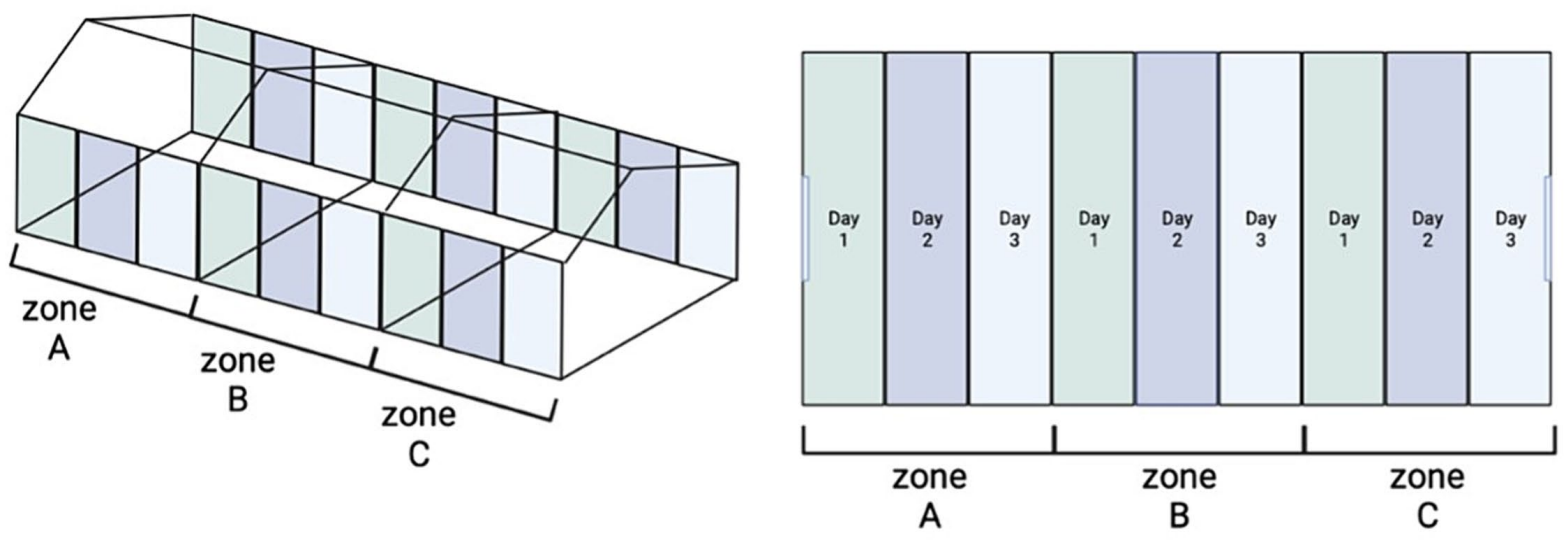
Scheme of the C&AP^2^&D sampling procedure. Figure on the left represents presents in an overall view how the houses are divided into 3 identical zones. The figure on the right shows in different color the zone which includes all sample type (floor, windows, walls, feeders, drinkers, and fans) each day of sampling.

All houses included in this study harbored persistent *Salmonella* bacteria despite undergoing the standard Cleaning and Disinfection (C&D) procedure. Thus, these farms were subjected to a modified C&D protocol which included an additional step which was the application of the AP twice consecutively at 24 h intervals (C&AP^2^&D). The standard C&D procedure comprised six steps: (i) initial cleaning to remove bedding, dust, and feces from the utensils within the facility (drinkers, feeders, etc.), followed by (ii) thorough rinsing with water, and (iii) Subsequent cleansing with detergent, rinsing to remove any detergent residue, and allowing the surface to dry before applying the disinfectant. A variety of disinfectants were utilized, with rotation based on the specific requirements of each facility. These included combinations of glutaraldehyde, quaternary ammonium compounds, and peroxides. The AP in each step was applied at a concentration of 10^8^PFU/mL. A total volume of 500 mL of AP diluted in 4.5 L of water was sprayed each time. The steps that were carried out were as follows: (i) Cleaning with detergent (removal of organic matter from the houses wet and dry), (ii) first application of the AP via spray, (iii) Second application of AP (24 h after the first application) and the last step, (iv) application of conventional disinfectants (24 h after the second AP application). To determine whether the houses were positive for *S*. Infantis, prior to the application of the modified procedure, swab samples from the poultry farms were collected according to the NSCP and analyzed following the ISO 6579:2017 (Mooijman et al., 2019; PNCS, 2020). After the application of each step (i. ii. and iii.) of the procedure C&AP^2^&D, representative samples from the floor, windows, walls, feeders, drinkers, and fans were taken from the houses. To this end, the farm was divided into 3 identical zones and each of these zones was divided into 3 parts (Figure 1). In this way on each of the sampling days, the surface area floor and walls were taken using boot swabs and swab cloths, respectively. In addition, swabs cloths were used to collect samples from the feeders, drinkers, and fans. To collect these samples, each of the surfaces to be sampled was divided into 3 parts, so that different areas were sampled without swabbing three times the same place. All these samples were analysed following the ISO 6579:2012 (ISO 6579:2012, 2012). Finally, after the last step (iv.) of the procedure C&AP^2^&D, samples were taken from the houses as laid by the NCSP to verify the absence of *Salmonella* (PNCS, 2020).

### 2.10 Statistical analysis

A Generalized Linear Model (GLM) was employed due to its flexibility in handling non-normally distributed data and due to the incorporation of multiple variables, providing a robust and flexible approach to assess the efficacy of AP in reducing *Salmonella* surfaces. To this end, a GLM was performed according to Sevilla-Navarro et al. (2020a). Concentrations (CFU/sample) of *Salmonella* counts were converted to Log_10_CFU/sample. Then, to evaluate the impact of the AP^2^ on *Salmonella* counts following each application, the GLM was fitted with the sampling moment (after cleaning, after 1st AP application, after 2nd AP application) as the response variable and the farm as the fixed effect. Additionally, another GLM was conducted to investigate the effect of each AP application time on *Salmonella* counts across various sample types, encompassing drinker, feeder, floor, fan, window, and wall. Here, the sampling moment served as the response variable, while the sample type was considered the fixed effect. A significance level of *p*-value < 0.05 was adopted to denote statistical significance in all analyses. Moreover, antibiotic resistance among *Salmonella* isolates was compared utilizing a GLM, wherein binomial data were coded as 1 for resistant isolates and 0 for non-resistant ones. The error distribution was specified as binomial, and the probit link function was applied. A significance threshold of *p*-value < 0.05 was utilized to determine statistical significance. All statistical analyses were performed using SPSS 27.0 software (SPSS Inc., Chicago, IL).

## 3 Results

### 3.1 Antimicrobial susceptibility test for the *Salmonella* isolates

A total of 10 farms were used in this study from which a total of 22 poultry houses were sampled. All *Salmonella* strains isolated from each farm were tested prior the C&AP^2^&D procedure. All of them (*n* = 22) were resistant to at least one of the 14 antimicrobials tested and 72.7 ± 9.7% (16/22) were MDR to three or more of the groups of antimicrobials. The highest percentages of AMR were found to be CIP (95.0 ± 4.4%, 21/22), and NAL (91.0 ± 6.1%, 20/22), followed by TMP and TET (68.0 ± 9.9%, 15/22, each) and SMX (64.0 ± 10.3%, 14/22), and finally AMP (14.0 ± 7.3%, 3/22), TGC (9.0 ± 6.1%, 2/22), and COL, FOT, GEN and TAZ (5.0 ± 4.4%, 1/22, each) (*p* < 0.05). Resistance to AZI, CHL, and MER was not observed. Overall, 6 different resistance patterns were observed. The combination of QNL-SULF-TE (59.1%, 13/22) was the most frequently observed pattern, followed by QNL alone (13.6%, 3/22), QNL-TE and QNL-B-LAC-SULF-TE (9.1%, 2/22, each), QNL-B-LAC-SULF-POL and B-LAC-AMG (4.5%, 1/22, each).

### 3.2 Characterization of *Salmonella* Infantis phages

At each farm, a phage capable of lysing its corresponding propagation strain was isolated. However, after sequencing analysis of the 10 isolated phages, a surprising similarity emerged among them, leading to the characterization of four distinct phages: vB_Si_CECAV_FGS009, vB_Si_CECAV_FGS017, vB_Si_CECAV_FGS029, and vB_Si_CECAV_FGS030.

### 3.3 Genomic analysis

MiSeq Illumina sequencing run led to 21,250–51,047 cleaned paired end reads that were used for generating the *de novo* assemblies with SPAdes. After removing bacterial contaminants and short contigs, one long assembly with high coverage was obtained for all the phages. BLAST comparison against the nucleotide database showed vB_Si_CECAV_FGS009, vB_Si_CECAV_FGS017 and vB_Si_CECAV_FGS029 were closed to phages belonging to *Tequintavirus* genus while vB_Si_CECAV_FGS030 was more related to phages belonging to the genera *Felixounavirus*. Enterobacteria phage T5 (Accesion Number: NC_005859) and *Salmonella* phage FelixO1 (Accesion Number: NC_005282.1), type-phages from *Tequintavirus* and *Felixounavirus* genus respectively, were used to rearrange the obtained genomes. Reordered assemblies were then corrected with Pilon leading to phage genome sizes bigger than 85 kb, and a GC of content around 39% in all the cases (Table 1).

**Table 1:** General characteristic of isolated phage genomes.

		vB_Si_CECAV_FGS009	vB_Si_CECAV_FGS017	vB_Si_CECAV_FGS029	vB_Si_CECAV_FGS030
Size		108,396	110,054	111,226	86,158
GC Content (%)		39.08	38.95	38.79	38.84
Average sequencing coverage		168,27	114,02	126,66	94,40
Coding sequences		202	200	205	144
tRNA and pseudogenes		23	23	24	24
Most similar by BLAST	Name	NC_028840.1 *Escherichia* phage slur09	MW006479.1 *Salmonella* phage GEC_vB_N5	NC_048627.1 *Escherichia* phage SP15	OQ174506.1 *Escherichia* phage REP7
	Length	111,751	110,015	110,964	88,978
	Coverage (%)	91	85	84	95
	E-value	0.0	0.0	0.0	0.0
	Identity (%)	96.94	95.89	95.22	95.70
Taxonomy	Class	*Caudoviricetes*	*Caudoviricetes*	*Caudoviricetes*	*Caudoviricetes*
	Family	*Demerecviridae*	*Demerecviridae*	*Demerecviridae*	*-*
	Genus	*Tequintavirus*	*Tequintavirus*	*Tequintavirus*	*Felixounavirus*

Features including genomic size, percentage of GC, sequencing coverage, predicted coding sequences, tRNAs and pseudogenes and most similar phage in nucleotide database by BLAST analysis are shown.

-: Taxonomic rank not determined by NCBI Taxonomy.

**Figure 2:**
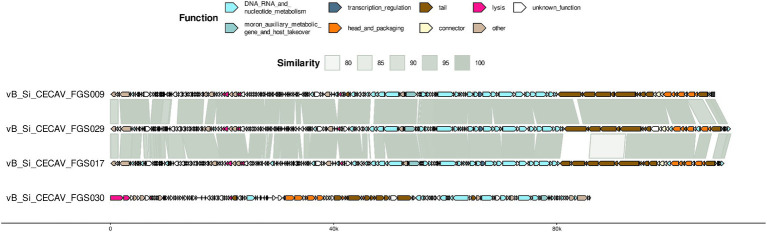
Genome organization and comparative genomics. For each phage, predicted coding sequences by Pharokka are represented as arrows colored based on the associated Phrog functional group. BLAST comparison throughout genomes is included.

Functional and structural annotation with Pharokka found at least 200 coding sequences in cecav_Si_CECAV_FGS009, cecav_Si_CECAV_FGS017 and cecav_Si_CECAV_FGS029 while 144 coding sequences were identified in phage cecav_Si_CECAV_FGS030 genome. In the four phages more than 200 tRNA and pseudogenes were also detected. Genomic organization of all phages was like other previously described phages belonging to *Tequintavirus* and *Felixounavirus* genus (Nicolas et al., 2023) (Figure 2).

Regarding antibiofilm properties of the phages, genetic markers were found. Pharokka annotation found 2 ORFs encoding a lysin in vB_Si_CECAV_FGS009, vB_Si_CECAV_FGS017 and vB_Si_CECAV_FGS029 genomes, and 1 ORF in vB_Si_CECAV_FGS030. Additionally, DePP results showed phage vB_Si_CECAV_FGS030 encoding one potential depolymerase (ORF present in the positive strand), while three potential depolymerases were found in vB_Si_CECAV_FGS017 and four in vB_Si_CECAV_FGS009 and vB_Si_CECAV_FGS029 (Table 2). All these predicted depolymerases were annotated as tail-related proteins by Pharokka. PhageLeads and PhaTYP analysis showed all four phages are strictly lytic (absence of temperate markers and probability of 1.0) and do not contain any sign of bacterial genome integration (absence of virulence and antimicrobial resistance genes).

**Table 2:** Predicted depolymerases in the phage genomes.

Phage	ORF	Start	End	Strand	Function	Product
vB_Si_CECAV_FGS009	173	80,206	84,282	−	Tail	Tail fiber protein
	175	84,710	86,812	−	Tail	Straight fiber tail protein
	176	86,813	89,662	−	Tail	Central tail fiber J
	196	105,230	106,990	−	Tail	Receptor binding tail protein
vB_Si_CECAV_FGS017	168	80,611	82,356	−	Tail	Tail fiber protein
	172	85,406	87,436	−	Tail	Straight fiber tail protein
	173	87,463	90,312	−	Tail	Central tail fiber J
vB_Si_CECAV_FGS029	174	81,379	85,275	−	Tail	Tail fiber protein
	176	85,702	87,804	−	Tail	Straight fiber tail protein
	177	87,805	90,654	−	Tail	Central tail fiber J
	199	107,597	109,384	−	Tail	Receptor binding tail protein
vB_Si_CECAV_FGS030	89	51,488	54,007	+	Tail	Tail fiber protein

Protein products with a predicted probability of at least 90% of being a depolymerase by DePP are shown. For each protein, information regarding ORF coordinates, strand and predicted function and product by Pharokka annotation is included.

**Figure 3:**
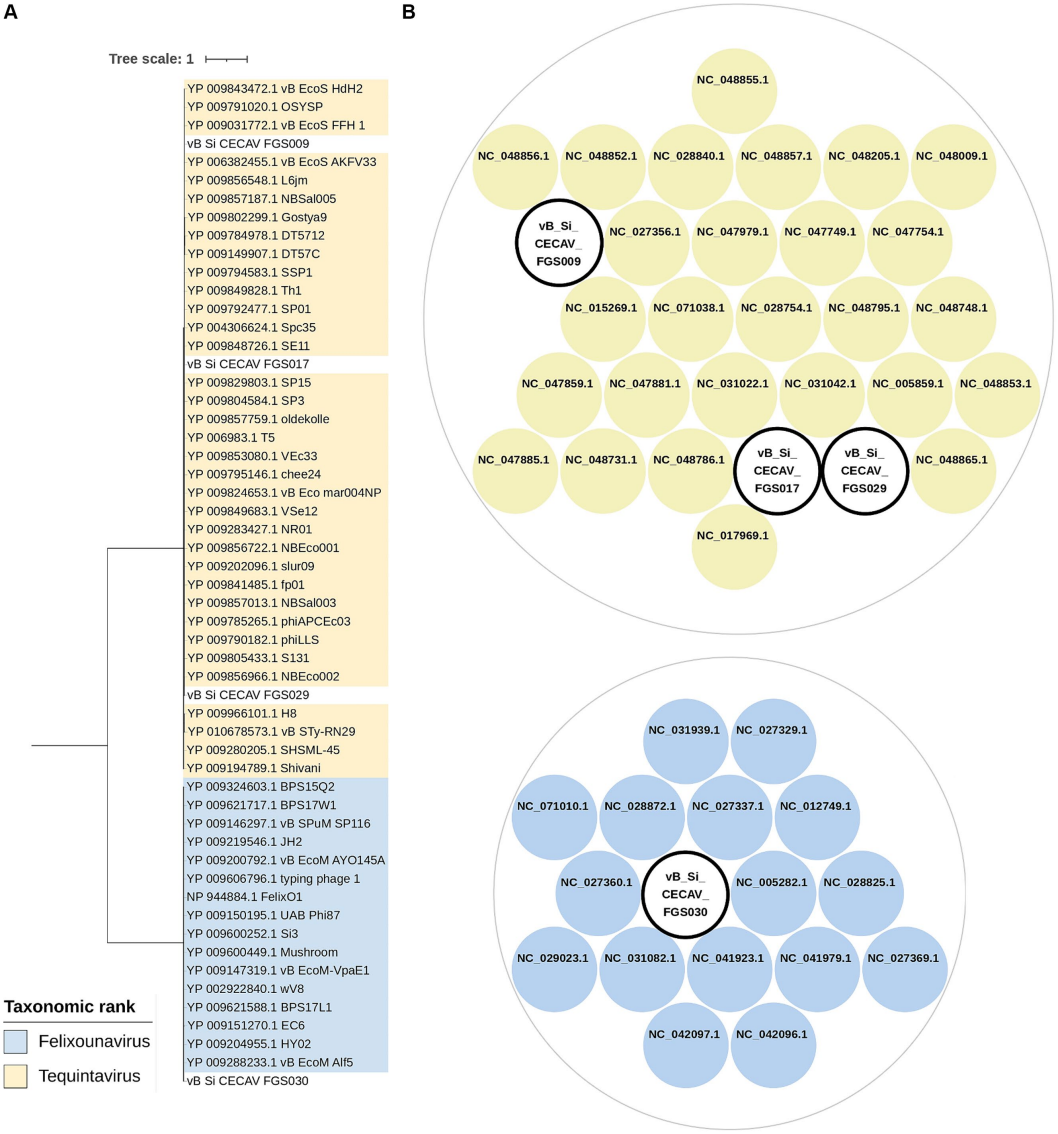
Taxonomic classification of isolated phages. **(A)** Maximum likelihood phylogenetic tree using terminase large subunit protein sequences. Midpoint was fixed as root and only branches with a bootstrap value of at least 90 are shown. **(B)** Clusters generated from intergenomic similarity calculation by VIRIDIC. Cut-off values for genus and species delimitation were fixed in 70 and 95%, respectively. Circle packaging graph was generated using RawGraphs.

Regarding taxonomic classification both whole-genome comparison and single-gene phylogeny using large terminase subunit sequence both analyses grouped vB_Si_CECAV_FGS009, vB_Si_CECAV_FGS017 and vB_Si_CECAV_FGS029 together with other phages belonging to the *Tequintavirus* genus (Figure 3). Specifically, vB_Si_CECAV_FGS009 and vB_Si_CECAV_FGS029 were more similar (88.1% of intergenomic similarity), while vB_Si_CECAV_FGS017 seemed to be slightly different (81.6 and 82.5% of similarity in comparison with vB_Si_CECAV_FGS009 and vB_Si_CECAV_FGS029, respectively). Interestingly, vB_Si_CECAV_FGS030 was more different in comparison with the other three isolated phages, and closer to phages from *Felixounavirus* genus (Figure 3).

### 3.4 Transmission electron micrographs of the isolated phages

**Figure 4:**
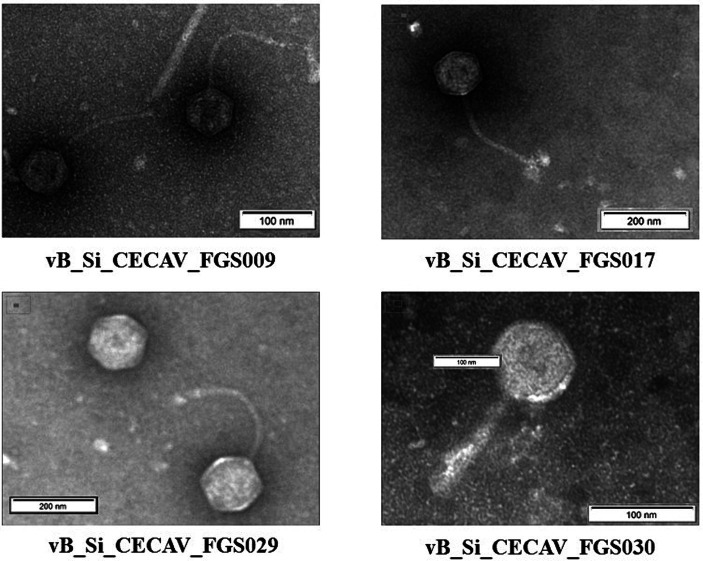
Electron transmission micrographs of the isolated *S*. Infantis phages.

The average head size of *Felixounavirus* phage was 80.7 nm, whereas for *Tequintavirus* phages the average head size ranged from 83.3 to 90.1 nm. The tail size of the *Felixounavirus* phage was 118.4 nm. However, it was 170.6–198.8 nm for *Tequintavirus* (Figure 4).

### 3.5 Determination of the phage host range and efficiency of plating (EOP)

**Figure 5:**
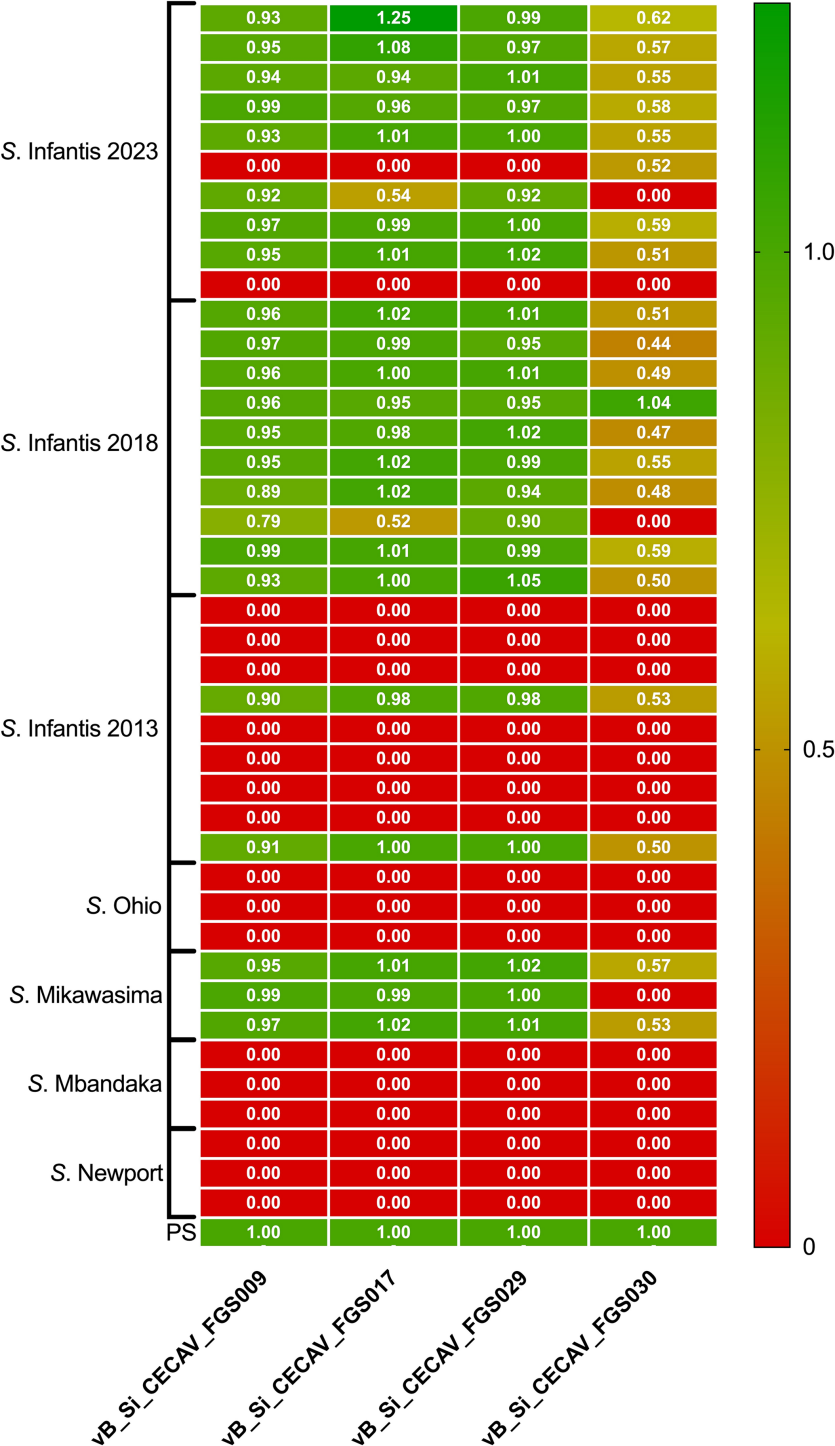
Heatmap of relative EOP for phages vB_Si_CECAV_FGS009; vB_Si_CECAV_FGS017; vB_Si_CECAV_FGS029 and vB_Si_CECAV_FGS030 for 41 *Salmonella* isolates. Values represent the average of three replicates. The EOP value for the phage-bacteria combination was classified as “highly virulent” for ratio > 0.5, “medium virulent” for ratios ≥ 0.1 and ≤ 0.5, “avirulent but active” for ratios >0.001 and < 0.1 and “inefficient” for ratios ≤0.001 or no plaques detected.

None of the phages demonstrated the ability to lyse serovars other than *S*. Infantis, except in the case of *S*. Mikawasima, where all phages, excluding vB_Si_CECAV_FGS030, exhibited high virulence against 100% of the isolates. Concerning *S*. Infantis from 2023 and 2018, phages vB_Si_CECAV _FGS009, vB_Si_CECAV_FGS018, and vB_Si_CECAV_FGS029 highly displayed virulence against 90% (18/20), while phage vB_Si_CECAV_FGS030 demonstrated highly and moderate virulence against 60 and 40% of isolates, respectively. Notably, there was a discernible difference in the infectivity among isolates from 2013, with all phages only able to infect a single strain (Figure 5).

### 3.6 Assessment of cleaning and disinfection procedure with AP in farms

Concerning the assessment of the cleaning and disinfection procedure with AP, statistically significant differences were found between the different steps of the C&AP^2^&D procedure (*p* < 0.001). Before the application of the phages, 100% of the houses tested positive for the presence of *S*. Infantis. Following the initial phage application, a reduction of 27% was observed, resulting in a positivity rate of 73%. After a second application, there was an additional reduction of 64%, bringing the positivity rate down to 36%. Ultimately, after the disinfection process, all houses tested negative for the presence of *S*. Infantis. Moreover, a noteworthy decline in bacterial presence was evident between procedure steps, leading to the complete elimination of bacteria when the initial count was below 5 logs. Table 3 illustrates the dynamic progression of *Salmonella* reduction in houses throughout the distinct stages of the C&AP^2^&D procedure.

**Table 3:** *Salmonella* Infantis reduction (Log_10_CFU/sample) in each step of the C&AP^2^&D procedure.

Farm	Pen	After cleaning	After 1st AP	After 2nd AP	After Disinfection	*p*-value*
1	1	9.65^a^	0.58^b^	0.28^b^	0^b^	**0.000**
	2	5.1^a^	0.78^b^	0.15^b^	0^b^	**<0.001**
2	3	0.62^a^	0^b^	0^b^	0^b^	0.261
3	4	1.94^a^	0^b^	0^b^	0^b^	**0.041**
	5	1.53^a^	0^b^	0^b^	0^b^	0.121
	6	1.39^a^	0^b^	0^b^	0^b^	0.121
4	7	5.03^a^	4.8^a^	0.21^b^	0^b^	**0.002**
	8	3.5^a^	1.4^ab^	0^b^	0^b^	**0.021**
5	9	1.21^a^	0^b^	0^b^	0^b^	0.121
	10	1.33^a^	0^b^	0^b^	0^b^	0.121
6	11	2.9^a^	2^ab^	0^b^	0^b^	**0.017**
	12	5.5^a^	4.5^b^	1.2^b^	0^b^	**<0.01**
	13	9.2^a^	5.9^b^	3.2^c^	0^d^	**<0.01**
7	14	9.55^a^	7.2^b^	3.33^c^	0^d^	**0.000**
8	15	4.7^a^	1.5^b^	0^b^	0^b^	**0.001**
	16	3.5^a^	0.33^b^	0^b^	0^b^	**<0.001**
	17	2.33^a^	1.55^a^	0^b^	0^b^	**0.094**
	18	4.9^a^	3.66^a^	0^b^	0^b^	**0.008**
9	19	3.75^a^	2.61^ab^	0.3^b^	0^b^	**0.017**
	20	3.25^a^	1.66^b^	0^b^	0^b^	**<0.001**
10	21	5.1^a^	1.25^b^	1^b^	0^b^	**<0.001**
	22	3.7^a^	1^b^	0^b^	0^b^	**<0.001**

*Bold values indicate statistically significant differences.

In relation to the type of sample tested for *Salmonella* presence, notable differences were observed post-cleaning step concerning *Salmonella* prevalence per sample (*p* = 0.011). The walls exhibited a higher prevalence of the bacterium (5.61 Log_10_CFU/sample), followed by the floor (5.22 Log_10_CFU/sample) and drinkers (3.66 Log_10_CFU/sample). However, after the 1st and 2nd application of the AP^2^, no significant differences were noted between samples. Conversely, significant variations emerged between the reduction of *Salmonella* in each sample and the sampling moment, with the most substantial reduction occurring after the second application of the AP, as outlined in Table 4.

**Table 4:** *Salmonella* enumeration in samples at each step of the C&AP^2^.

Sample	After Cleaning	After 1^st^ AP	After 2^nd^ AP	*p*-value
Drinker	3.66^a, A,B,C^	1.84^a^	0.73^b^	0.009
Feeder	3.22^a, A, C^	1.40^b^	0.14^b^	0.001
Floor	5.22^a, A, B^	1.88^b^	0.30^b^	0.032
Fan	2.22 ^a, C^	0.66^b^	0.21^b^	0.003
Window	2.84 ^a, C^	1.16^b^	0.22^b^	0.006
Wall	5.61 ^a, B^	2.63^b^	0.45^c^	0.003
*p*-value	0.011	0.941	0.508	

^a,b,c^ different superscripts means significant differences between rows with a *p*-value < 0.05; ^A,B,C^ different superscripts means significant differences between columns with a *p*-value < 0.05.

## 4 Discussion

In this study, the use of phages as an intermediate step between cleaning and disinfection resulted in a statistically significant decrease in the number of farms testing positive for *S*. Infantis. To the best of our knowledge, this is the first published study investigating the efficacy of phages in combination with cleaning and disinfection procedures against *S*. Infantis in real-world field conditions.

Foodborne pathogens that survive cleaning and disinfection during poultry processing, are a public health concern due to the increased possibility of spreading to humans and mobilizing antibiotic resistance (Cadena et al., 2019). Unlike other *Salmonella* serovars that are controlled in one or a few flock cycles, *S*. Infantis has been shown to be more persistent and difficult to remove from poultry house surfaces, mainly due to biofilm formation (Drauch et al., 2020; Bezek et al., 2023). This indicates that *S*. Infantis might be more resistant to standard cleaning and disinfection protocols. In this regard, the need to control bacteria on the farm, as well as the emergence of MDR bacteria, has encouraged many researchers to investigate new alternative biocontrol agents, such as the use of phages.

Currently, there are no specific measures to control *S*. Infantis on farms. Other *Salmonella* serovars such as *S*. Enteritidis and *S*. Typhimurium have reduced their incidence due to the use of live vaccines (Fiorino et al., 2017). In this line, the use of *S*. Infantis vaccines has been studied, either as a serovar-specific vaccine or for cross immunization across *Salmonella* serovars (Eeckhaut et al., 2018; Crouch et al., 2020a,b; Sáenz et al., 2022), showing promising results in the reduction of *S*. Infantis fecal shedding and internal colonization in both layer and breeder hens (Crouch et al., 2020a,b). Nevertheless, the current problem with *S*. Infantis is mainly related to broilers, and there are currently no commercial live vaccines available against *S*. Infantis in broilers (Sáenz et al., 2022). Thus, *S*. Infantis incidence in poultry is mainly controlled by biosecurity measures, C&D protocols and good management practices. For this reason, efforts to control *S*. Infantis in poultry farms have intensified in recent years. Phages are a natural biocontrol measure, using microorganisms—viruses, in this case—to combat other microorganisms (Gildea et al., 2022). Numerous studies have demonstrated the effectiveness of *Salmonella* phages in reducing *Salmonella* levels within the poultry industry. These investigations have revealed significant reductions in *Salmonella* colonization in the gastrointestinal tract of chickens, while simultaneously ensuring their health and well-being (Lavilla et al., 2023). Additionally, research has explored the use of phages as a disinfectant to eliminate *Salmonella* from poultry drinkers, a critical point for *Salmonella* cross-infection (Korzeniowski et al., 2022), as well as from cement floors where *Salmonella* exhibits high resistance to conventional disinfection methods (Sevilla-Navarro et al., 2020a). The acceptance of phage therapy is gaining traction as an innovative and environmentally friendly approach that could potentially replace or enhance conventional methods for controlling pathogens, such as chemical disinfectants and antimicrobials (Cristobal-Cueto et al., 2021; Vikram et al., 2021; Gildea et al., 2022).

Here, *S*. Infantis isolates were obtained from poultry farms revealing a concerning trend, all isolates were AMR, and >72.7 were MDR. High levels of resistance to CIP, NAL, TMP and TET were observed. This resistance has been linked to the presence of the pESI mega-plasmid (Mughini-Gras et al., 2021). *S*. Infantis isolates carrying pESI not only exhibited high levels of AMR but also an increased ability to form biofilms, contributing to the persistence of *Salmonella* in poultry facilities (Alba et al., 2020; Mughini-Gras et al., 2021). Given the role of biofilms in tolerance and resistance, new treatments are needed, and phages present an intriguing alternative (Ferriol-González and Domingo-Calap, 2020; Erol and Kaskatepe, 2024).

The four phages examined in this study (vB_Si_CECAV_FGS009, vB_Si_CECAV_FGS017, vB_Si_CECAV_FGS029, vB_Si_CECAV_FGS030) demonstrated strict lytic behavior and multiple depolymerase genes, highlighting their potential as efficient biocontrol agents. These features make them potentially effective against both biofilm and antibiotic-resistant bacteria (Hibstu et al., 2022). Furthermore, the host range of these phages has demonstrated high specificity for the Infantis serovar, showcasing a broad yet selective host range. They effectively lysed different strains within the Infantis serovar, including the Mikawasima serovar. An intriguing observation within the host range is the predominant lysis of strains from 2018 and 2023, while strains from 2013 resisted lysis. This prompts suspicion that *S*. Infantis strains may have undergone evolutionary changes, aligning with previous reports of such evolution in other countries over the years (Alvarez et al., 2023). Considering the global emergence of multidrug-resistant (MDR) *S*. Infantis and its adaptive potential over time, it becomes imperative to consider these factors when formulating a resilient strategy (Ross et al., 2016). While studies have demonstrated the efficacy of phages as sanitizers in the food industry, this study is the first to evaluate their effectiveness in commercial poultry farms in combination with cleaning and disinfection protocols. For this purpose, a standardized cleaning and disinfection protocol was tested together with two consecutive field-level AP applications, the C&AP^2^&D procedure, as a methodology to prevent *S*. Infantis food-borne infections. The results of this study show that two phage applications in the C&D procedure significantly reduced the contamination of the poultry farms as when C&AP^2^&D was applied in the field, *Salmonella* was reduced from 100% after cleaning to 36% after the AP application, and 0% after disinfection, eliminating the *Salmonella* completely from the farm facilities. This complete elimination of *Salmonella* from the farm facilities is noteworthy, especially considering that all farms subjected to the study tested positive following conventional cleaning and disinfection procedures. These findings suggest that the synergistic application of C&D protocols and bacteriophage treatments holds the potential for achieving a higher level of bacterial reduction compared to each treatment in isolation.

One of the main issues regarding phage therapy is the possible emergence of phage-resistant bacterial variants, which could impede favorable outcomes on a long-term basis (Oechslin, 2018). To reduce the effects such variants, have on phage therapy, in-depth characterizations of the bacteriophages and cocktails of different phages should be carried (Leskinen et al., 2017; Aguilera et al., 2023). Nevertheless, the isolation of AP from poultry farms, could also bypass this issue, since the simultaneous presence of both bacteria and phage in the same environment for long periods of time leads to co-evolution dynamics (Olszak et al., 2017; Sevilla-Navarro et al., 2018; Torres-Boncompte et al., 2023). Co-evolution dynamics occur when, through spontaneous mutations, host bacteria develop resistance mechanisms against the bacteriophage to reduce their lytic ability and bacteriophages adapt to such changes to maintain their lytic capacities upon their host (Oechslin, 2018). Therefore, the repeated isolation of an AP from the same facilities spared in time could minimize the risk bacterial variants represent for phage therapy.

The variability observed in the removal of bacteria from the farm facilities, achieved through either a single or double application, as opposed to others that exhibited persistence until the post-disinfection stage, may be attributed to the reported differences in disinfectant susceptibility among *S*. Infantis strains or variations in the initial level of *Salmonella* contamination ranging from 9.65 Log_10_CFU/sample to 0.62 Log_10_CFU/sample (Drauch et al., 2020). Furthermore, the effectiveness of the C&AP^2^&D method showed variability in reducing *Salmonella* levels across different steps of the procedure, highlighting the necessity of implementing two consecutive bacteriophage applications to achieve maximal reduction. These findings align with previous studies, indicating that successive phage applications were essential for optimal *Salmonella* reduction in both controlled experiments and a commercial layer farm, despite the occurrence of phage auto-amplification *in situ* (Sevilla-Navarro et al., 2018, 2020a). Concerning the type of sample tested, significant differences were found in those collected after the cleaning step, with higher levels of contamination remaining in *Salmonella* floor, drinkers, and wall samples. This is in accordance with previous studies that have demonstrated that drinker cups, drain holes in the walls and floor cracks are critical locations during the C&D procedure, being an early re-exposure of *Salmonella* to new flocks (Luyckx et al., 2015; Martelli et al., 2017).

Phage biocontrol has been widely demonstrated as a potential tool against AMR pathogens and as a sanitizer in the food industry (Gutiérrez et al., 2016; Brives and Pourraz, 2020). However, less attention has been paid to the possibility of using phages as a biocontrol agent in livestock and the environment (Stachler et al., 2021). The absence of an ecological impact on the environment of introducing phages into the farm environment should be considered. The application of specific phages, in this case, phages against *S*. Infantis, might not represent a threat to other bacteria (Gill and Hyman, 2010). The high host specificity of phages against their target bacterium might prevent further alterations in the environmental bacterial communities or in the microbiota of the animals to be reared in the facilities (Clokie et al., 2011; Gildea et al., 2022). Even so, in this study, phages were inactivated after the application of the disinfectant. In this way, not only were the current C&D regulations complied with, but also the phages were inactivated before their release outside the industry environment (Gutiérrez et al., 2016), despite the use of AP isolated from each treated farm. Thus, this type of application could be described as environmentally friendly since no new microorganisms are introduced (Sevilla-Navarro et al., 2018). The present study gives insight into the potential use of phages in combination with the current C&D protocols as a strategy to reduce persistent *S*. Infantis strains from the field, as after the application of AP^2^ significant reductions were observed, reaching levels close to zero and achieving complete absence after disinfection. However, further studies are needed to demonstrate phage biocontrol in combination with different disinfectant substances to eradicate bacterial biofilms and their impact on the environmental microbiota.

## 5 Conclusion

Overall, this study demonstrates the efficacy of phages as disinfectants on poultry farms to prevent a major threat of food-borne pathogens. Interestingly, the C&AP^2^&D procedure described here reduced drastically the presence of *Salmonella* on farms, successfully eliminating the bacteria, in contrast to conventional C&D protocols. The ability of phages removing bacterial biofilms in the field should be tested on other bacterial species with zoonotic potential and will help reduce the spread of MDR bacteria worldwide. Therefore, this cost-effective and environmentally sustainable tool should be considered as a promising alternative available to eliminate pathogenic bacteria from the farm facilities. Despite being challenging, “One Health” approaches should be in the spotlight to control a major concern and to develop novel economic and eco-friendly strategies, with phages being a promising preventive tool against MDR bacteria.

## Data availability statement

The datasets presented in this study can be found in online repositories. The names of the repository/repositories and accession number(s) can be found here: https://www.ncbi.nlm.nih.gov/genbank/, accession numbers: PP407513, PP429239, PP429240, PP429241.

## Author contributions

SS-N: Conceptualization, Data curation, Formal analysis, Funding acquisition, Investigation, Methodology, Project administration, Resources, Software, Supervision, Validation, Visualization, Writing – original draft, Writing – review & editing. JT-B: Data curation, Writing – original draft, Writing – review & editing. JG-L: Writing – review & editing, Methodology. MB-G: Formal analysis, Writing – review & editing. PD-C: Funding acquisition, Writing – review & editing. PC-G: Conceptualization, Methodology, Supervision, Validation, Writing – review & editing.

## References

[ref1] Abedon S. T. Danis-Wlodarczyk K. M. Wozniak D. J. (2021). Phage cocktail development for bacteriophage therapy: toward improving Spectrum of activity breadth and depth. Pharmaceuticals 14:1019. doi: 10.3390/PH14101019 34681243 PMC8541335

[ref2] Aguilera M. Tobar-Calfucoy E. Rojas-Martínez V. Norambuena R. Serrano M. J. Cifuentes O.. (2023). Development and characterization of a bacteriophage cocktail with high lytic efficacy against field-isolated *Salmonella enterica*. Poult. Sci. 102:103125. doi: 10.1016/j.psj.2023.103125 37879168 PMC10618821

[ref3] Alba P. Leekitcharoenphon P. Carfora V. Amoruso R. Cordaro G. Di Matteo P.. (2020). Molecular epidemiology of Salmonella Infantis in Europe: insights into the success of the bacterial host and its parasitic pESI-like megaplasmid. Microb. Genom. 6, 1–12. doi: 10.1099/MGEN.0.000365 PMC7371121 32271142

[ref4] Altschul S. F. Gish W. Miller W. Myers E. W. Lipman D. J. (1990). Basic local alignment search tool. J. Mol. Biol. 215, 403–410. doi: 10.1016/S0022-2836(05)80360-2 2231712

[ref5] Alvarez D. M. Barrón-Montenegro R. Conejeros J. Rivera D. Undurraga E. A. Moreno-Switt A. I. (2023). A review of the global emergence of multidrug-resistant *Salmonella enterica* subsp. enterica Serovar Infantis. Int. J. Food Microbiol. 403:110297. doi: 10.1016/J.IJFOODMICRO.2023.110297 37406596

[ref6] Bankevich A. Nurk S. Antipov D. Gurevich A. A. Dvorkin M. Kulikov A. S.. (2012). SPAdes: a new genome assembly algorithm and its applications to single-cell sequencing. J. Comput. Biol. 19, 455–477. doi: 10.1089/cmb.2012.0021 22506599 PMC3342519

[ref7] Bezek K. Avberšek J. Zorman Rojs O. Barlič-Maganja D. (2023). Antimicrobial and Antibiofilm effect of commonly used disinfectants on Salmonella Infantis isolates. Microorganisms 11:301. doi: 10.3390/MICROORGANISMS11020301 36838265 PMC9958858

[ref8] Bouras G. Nepal R. Houtak G. Psaltis A. J. Wormald P. J. Vreugde S. (2023). Pharokka: a fast scalable bacteriophage annotation tool. Bioinformatics 39:btac776. doi: 10.1093/bioinformatics/btac776 36453861 PMC9805569

[ref9] Brives C. Pourraz J. (2020). Phage therapy as a potential solution in the fight against AMR: obstacles and possible futures. Palgrave Commun. 6, 1–11. doi: 10.1057/s41599-020-0478-4

[ref10] Bushnell B. (2014). BBMap: a fast, accurate, splice-aware aligner. Berkeley, CA: Lawrence Berkeley National Lab.

[ref11] Cadena M. Kelman T. Marco M. L. Pitesky M. (2019). Understanding antimicrobial resistance (AMR) profiles of Salmonella biofilm and planktonic Bacteria challenged with disinfectants commonly used during poultry processing. Food Secur. 8:275. doi: 10.3390/FOODS8070275 PMC6678331 31336660

[ref12] Clinical and Laboratory Standards Institute (2024). Performance Standards for Antimicrobial Susceptibility Testing. United States: Clinical & Laboratory Standards Institute.

[ref13] Clokie M. R. Millard A. D. Letarov A. V. Heaphy S. (2011). Phages in nature. Bacteriophage 1, 31–45. doi: 10.4161/bact.1.1.14942 21687533 PMC3109452

[ref14] Commission Implementing Decision (2020). Official Journal of the European Union. Available at: https://eur-lex.europa.eu/legal-content/EN/TXT/PDF/?uri=CELEX:32020D1729

[ref15] Corcoran M. Morris D. De Lappe N. O’connor J. Lalor P. Dockery P.. (2014). Commonly used disinfectants fail to eradicate *Salmonella enterica* biofilms from food contact surface materials. Appl. Environ. Microbiol. 80, 1507–1514. doi: 10.1128/AEM.03109-13 24362427 PMC3911063

[ref16] Cristobal-Cueto P. García-Quintanilla A. Esteban J. García-Quintanilla M. (2021). Phages in food industry biocontrol and bioremediation. Antibiotics 10:786. doi: 10.3390/antibiotics10070786 34203362 PMC8300737

[ref17] Crouch C. F. Nell T. Reijnders M. Donkers T. Pugh C. Patel A.. (2020b). Safety and efficacy of a novel inactivated trivalent *Salmonella enterica* vaccine in chickens. Vaccine 38, 6741–6750. doi: 10.1016/j.vaccine.2020.08.033 32888739

[ref18] Crouch C. F. Pugh C. Patel A. Brink H. Wharmby C. Watts A.. (2020a). Reduction in intestinal colonization and invasion of internal organs after challenge by homologous and heterologous serovars of *Salmonella enterica* following vaccination of chickens with a novel trivalent inactivated *Salmonella* vaccine. Avian Pathol. 49, 666–677. doi: 10.1080/03079457.2020.1814200 32907345

[ref01] Davis M. A. Hancock D. D. Besser T. E. (2002). Multiresistant clones of Salmonella enterica: the importance of dissemination. J. Lab. Clin. Med. 140, 135–41. doi: 10.1067/mlc.2002.126411 12271270

[ref19] D’accolti M. Soffritti I. Mazzacane S. Caselli E. (2021). Bacteriophages as a potential 360-degree pathogen control strategy. Microorganisms 9, 1–15. doi: 10.3390/MICROORGANISMS9020261 PMC7911525 33513949

[ref20] Drauch V. Ibesich C. Vogl C. Hess M. Hess C. (2020). In-vitro testing of bacteriostatic and bactericidal efficacy of commercial disinfectants against Salmonella Infantis reveals substantial differences between products and bacterial strains. Int. J. Food Microbiol. 328:108660. doi: 10.1016/J.IJFOODMICRO.2020.108660 32450393

[ref02] ECDC (2016). Surveillance of antimicrobial resistance in Europe 2016. Surveillance report.

[ref21] Eeckhaut V. Haesebrouck F. Ducatelle R. van Immerseel F. (2018). Oral vaccination with a live *Salmonella* Enteritidis/typhimurium bivalent vaccine in layers induces cross-protection against caecal and internal organ colonization by a *Salmonella* Infantis strain. Vet. Microbiol. 218, 7–12. doi: 10.1016/j.vetmic.2018.03.022 29685223

[ref22] EFSA and ECDC (2021). The European Union one health 2020 Zoonoses report. EFSA J. 19:e06971. doi: 10.2903/j.efsa.2021.6971 36329690 PMC9624447

[ref23] EFSA and ECDC (2023). The European Union one health 2022 Zoonoses report. EFSA J. 2023:E8442. doi: 10.2903/j.efsa.2023.8442 PMC10714251 38089471

[ref24] Erol H. B. Kaskatepe B. (2024). Effect of phage and rhamnolipid on Salmonella Infantis biofilm removal and biological control of phage on food deterioration. Int. J. Food Sci. Technol. 59, 120–128. doi: 10.1111/IJFS.16781

[ref25] Federal Register (2006). Food additives permitted for direct addition to food for human consumption; bacteriophage preparation Available at: https://www.federalregister.gov/documents/2006/08/18/E6-13621/food-additives-permitted-for-direct-addition-to-food-for-human-consumption-bacteriophage-preparation. 27101640

[ref26] Ferriol-González C. Domingo-Calap P. (2020). Phages for biofilm removal. Antibiotics 9:268. doi: 10.3390/ANTIBIOTICS9050268 32455536 PMC7277876

[ref27] Fiorino F. Rondini S. Micoli F. Lanzilao L. Alfini R. Mancini F.. (2017). Immunogenicity of a bivalent Adjuvanted Glycoconjugate vaccine against *Salmonella* typhimurium and *Salmonella Enteritidis*. Front. Immunol. 27:168. doi: 10.3389/fimmu.2017.00168 PMC5326758 28289411

[ref28] García-Soto S. Abdel-Glil M. Y. Tomaso H. Linde J. Methner U. (2020). Emergence of multidrug-resistant *Salmonella enterica* subspecies enterica Serovar Infantis of multilocus sequence type 2283 in German broiler farms. Front. Microbiol. 11:547030. doi: 10.3389/FMICB.2020.01741/BIBTEX PMC7380084 32765483

[ref03] Gibson S. B. Green S. I. Liu C. G. Salazar K. C. Clark J. R. Terwilliger A. L.. (2019). Constructing and characterizing bacteriophage libraries for phage therapy of human infections. Front. Microbiol. 10:2537. doi: 10.3389/fmicb.2019.02537 31781060 PMC6861333

[ref29] Gildea G. Ayariga J. A. Robertson B. K. (2022). Bacteriophages as biocontrol agents in livestock food production. Microorganism 10:2116. doi: 10.3390/microorganisms10112126 PMC9692620 36363718

[ref30] Gill J. J. Hyman P. (2010). Phage choice, isolation, and preparation for phage therapy. Curr. Pharm. Biotechnol. 11, 2–14. doi: 10.2174/138920110790725311 20214604

[ref04] Gradel K. O. Randall L. Sayers A. R. Davies R. H. (2005). Possible associations between Salmonella persistence in poultry houses and resistance to commonly used disinfectants and a putative role of mar. Vet. Microbiol. 107, 127–38. doi: 10.1016/j.vetmic.2005.01.013 15795084

[ref31] Grimont P. A. D. Weill F.-X. (2007). Antigenic formulae of the salmonella serovars. Paris: WHO Collaborating Centre for Reference and Research on Salmonella.

[ref32] Gu D. Wang Z. Tian Y. Kang X. Meng C. Chen X.. (2020). Prevalence of Salmonella isolates and their distribution based on whole-genome sequence in a chicken slaughterhouse in Jiangsu, China. Front. Vet. Sci. 7:506245. doi: 10.3389/FVETS.2020.00029/BIBTEX PMC7046563 32154275

[ref33] Gutiérrez D. Rodríguez-Rubio L. Martínez B. Rodríguez A. García P. (2016). Bacteriophages as weapons against bacterial biofilms in the food industry. Front. Microbiol. 7:825. doi: 10.3389/FMICB.2016.00825 27375566 PMC4897796

[ref34] Hackl T. Ankenbrand M. van Adrichem B. (2023). Gggenomes: a grammar of graphics for comparative genomics. Available at: https://github.com/thackl/gggenomes.

[ref35] Hibstu Z. Belew H. Akelew Y. Mengist H. M. (2022). Phage therapy: A different approach to fight bacterial infections. Biologics 6, 173–186. doi: 10.2147/BTT.S381237 PMC9550173 36225325

[ref36] ISO 6579-2:2012 (2012). Microbiology of the food chain -Horizontal method for the detection, enumeration and serotyping of Salmonella - Part 2: Enumeration by miniaturized most probable number technique. Available at: https://standards.iteh.ai/catalog/standards/sist/0bbcfcc4-106a-47be-a7bf-a693b76e9451/iso-ts-6579-2-2012

[ref37] Korzeniowski P. Śliwka P. Kuczkowski M. Mišić D. Milcarz A. Kuźmińska-Bajor M. (2022). Bacteriophage cocktail can effectively control Salmonella biofilm in poultry housing. Front. Microbiol. 29:901770. doi: 10.3389/fmicb.2022.901770 PMC9277115 35847069

[ref38] Lavilla M. Domingo-Calap P. Sevilla-Navarro S. Lasagabaster A. (2023). Natural killers: opportunities and challenges for the use of bacteriophages in microbial food Safery from the one health perspective. Food Secur. 12:552. doi: 10.3390/foods12030552 PMC9914193 36766081

[ref39] Leskinen K. Tuomala H. Wicklund A. Horsma-Heikkinen J. Kuusela P. Skurnik M.. (2017). Characterization of vB_SauM-fRuSau02, a Twort-like bacteriophage isolated from a therapeutic phage cocktail. Viruses 14:258. doi: 10.3390/v9090258 PMC5618024 28906479

[ref40] Letunic I. Bork P. (2021). Interactive tree of life (iTOL) v5: an online tool for phylogenetic tree display and annotation. Nucleic Acids Res. 49, W293–W296. doi: 10.1093/nar/gkab301 33885785 PMC8265157

[ref41] Luyckx K. Y. Van Weyenberg S. Dewulf J. Herman L. Zoons J. Vervaet E.. (2015). On-farm comparisons of different cleaning protocols in broiler houses. Poult. Sci. 94, 1986–1993. doi: 10.3382/PS/PEV143 26047671

[ref42] Maes S. Vackier T. Nguyen Huu S.. (2019). Occurrence and characterisation of biofilms in drinking water systems of broiler houses. BMC Microbiol. 19:77. doi: 10.1186/s12866-019-1451-5 30987581 PMC6466764

[ref43] Magill D. J. Skvortsov T. A. (2023). DePolymerase predictor (DePP): a machine learning tool for the targeted identification of phage depolymerases. Bioinformatics 24:208. doi: 10.1186/s12859-023-05341-w 37208612 PMC10199479

[ref44] Martelli F. Gosling R. J. Callaby R. Davies R. (2017). Observations on Salmonella contamination of commercial duck farms before and after cleaning and disinfection. Avian Pathol. 46, 131–137. doi: 10.1080/03079457.2016.1223835 27545288

[ref45] Mauri M. Elli T. Caviglia G. Uboldi G. Azzi M. (2017). RAWGraphs: a visualisation platform to create open outputs. In Proceedings of the 12th Biannual Conference on Italian SIGCHI Chapter. ACM, New York, NY, USA.

[ref46] Mooijman K. A. Pielaat A. AFA K. (2019). Validation of EN ISO 6579-1 - Microbiology of the food chain - Horizontal method for the detection, enumeration and serotyping of Salmonella - Part 1 detection of Salmonella spp. Int. J. Food Microbiol. 288, 3–12. doi: 10.1016/j.ijfoodmicro.2018.03.022 29803313

[ref47] Moraru C. Varsani A. Kropinski A. M. (2020). VIRIDIC—a novel tool to calculate the intergenomic similarities of prokaryote-infecting viruses. Viruses 12:1268. doi: 10.3390/v12111268 33172115 PMC7694805

[ref48] Mughini-Gras L. van Hoek A. H. A. M. Cuperus T. Dam-Deisz C. van Overbeek W. van den Beld M.. (2021). Prevalence, risk factors and genetic traits of Salmonella Infantis in Dutch broiler flocks. Vet. Microbiol. 258:109120. doi: 10.1016/j.vetmic.2021.109120 34020175

[ref49] Murray C. J. Ikuta K. S. Sharara F. Swetschinski L. Robles Aguilar G. Gray A.. (2022). Global burden of bacterial antimicrobial resistance in 2019: a systematic analysis. Lancet 399, 629–655. doi: 10.1016/S0140-6736(21)02724-0 35065702 PMC8841637

[ref50] Newton K. Gosling B. Rabie A. Davies R. (2020). Field investigations of multidrug-resistant Salmonella Infantis epidemic strain incursions into broiler flocks in England and Wales. Avian Pathol. 49, 631–641. doi: 10.1080/03079457.2020.1809634 32783749

[ref51] Nicolas M. Trotereau A. Culot A. Moodley A. Atterbury R. Wagemans J.. (2023). Isolation and characterization of a novel phage collection against avian-pathogenic *Escherichia coli*. Microbiol. Spectr. 11:e0429622. doi: 10.1128/spectrum.04296-22 37140373 PMC10269720

[ref52] O’Neill J. (2014). Antimicrobial resistance: tackling a crisis for the health and wealth of nations. Available at: https://www.who.int/news/item/29-04-2019-new-report-calls-for-urgent-action-to-avert-antimicrobial-resistance-crisis

[ref53] O’Neill J. (2016). Tackling drug-resistant infections globally: final report and recommendations. London, United Kingdom: Review on Antimicrobial Resistance.

[ref54] Oechslin F. (2018). Resistance development to bacteriophages occurring during bacteriophage therapy. Viruses 10:351. doi: 10.3390/v10070351 29966329 PMC6070868

[ref55] Olszak T. Roszniowski B. Latka A. Valvano M. A. Drullis-Kawa Z. (2017). Phage life cycles behind bacterial biodiversity. Curr. Med. Chem. 24, 3987–4001. doi: 10.2174/0929867324666170413100136 28412903

[ref56] Pang X. Hu X. Du X.. (2023). Biofilm formation in food processing plants and novel control strategies to combat resistant biofilms: the case of *Salmonella* spp. Food Sci. Biotechnol. 32, 1703–1718. doi: 10.1007/s10068-023-01349-3 37780596 PMC10533767

[ref57] PNCS (2020). Programa Nacional de Control de Determinados Serotipos de Salmonella en la especie Gallus gallus. Madrid: PNCS.

[ref58] Ross A. Ward S. Hyman P. (2016). More is better: selecting for broad host range bacteriophages. Front. Microbiol. 7:1352. doi: 10.3389/FMICB.2016.01352 27660623 PMC5014875

[ref59] Sáenz L. Guzmán M. Vidal S. Caruffo M. Siel D. Zayas C.. (2022). Efficacy of multivalent, Cochleate-based vaccine against *Salmonella* Infantis, *S*. Enteritidis and *S.* typhimurium in laying hens. Vaccine 10:226. doi: 10.3390/vaccines10020226 PMC8879397 35214684

[ref60] Sevilla-Navarro S. Catalá-Gregori P. García C. Cortés V. Marin C. (2020a). *Salmonella* Infantis and *Salmonella* Enteritidis specific bacteriophages isolated form poultry faeces as a complementary tool for cleaning and disinfection against Salmonella. Comp. Immunol. Microbiol. Infect. Dis. 68:101405. doi: 10.1016/j.cimid.2019.101405 31887484

[ref61] Sevilla-Navarro S. Catalá-Gregori P. Marin C. (2020b). Salmonella bacteriophage diversity according to most prevalent Salmonella serovars in layer and broiler poultry farms from eastern Spain. Animals 10:1456. doi: 10.3390/ani10091456 32825110 PMC7552790

[ref62] Sevilla-Navarro S. Marín C. Cortés V. García C. Vega S. Catalá-Gregori P. (2018). Autophage as a control measure for Salmonella in laying hens. Poult. Sci. 97, 4367–4373. doi: 10.3382/ps/pey294 29982828

[ref63] Shang J. Tang X. Sun Y. (2023). PhaTYP: predicting the lifestyle for bacteriophages using BERT. Brief. Bioinform. 24:bbac487. doi: 10.1093/bib/bbac487 36659812 PMC9851330

[ref64] Silva C. Calva E. Maloy S. (2014). One health and food-borne disease: *Salmonella* transmission between humans, animals, and plants. Microbiol. Spectr. 2:OH-0020-2013. doi: 10.1128/MICROBIOLSPEC.OH-0020-2013 26082128

[ref65] Stachler E. Kull A. Julian T. R. (2021). Bacteriophage treatment before chemical disinfection can enhance removal of plastic-surface-associated *Pseudomonas aeruginosa*. Appl. Environ. Microbiol. 87, 1–12. doi: 10.1128/AEM.00980-21 PMC8478462 34347517

[ref66] Steenackers H. Hermans K. Vanderleyden J. De Keersmaecker S. C. J. (2012). Salmonella biofilms: an overview on occurrence, structure, regulation and eradication. Food Res. Int. 45, 502–531. doi: 10.1016/J.FOODRES.2011.01.038

[ref67] Szafrański S. P. Winkel A. Stiesch M. (2017). The use of bacteriophages to biocontrol oral biofilms. J. Biotechnol. 250, 29–44. doi: 10.1016/J.JBIOTEC.2017.01.002 28108235

[ref68] Thanki A. M. Hooton S. Gigante A. M. Atterbury R. J. Clokie M. R. J. Thanki A. M.. (2021). “Potential roles for bacteriophages in reducing Salmonella from poultry and swine” in Salmonella spp. - A Global Challenge. eds. Lamas A. Regal P. Franco C. M. (London: IntechOpen).

[ref69] Thompson J. D. Higgins D. G. Gibson T. J. (1994). CLUSTAL W: improving the sensitivity of progressive multiple sequence alignment through sequence weighting, position-specific gap penalties and weight matrix choice. Nucleic Acids Res. 22, 4673–4680. doi: 10.1093/nar/22.22.4673 7984417 PMC308517

[ref70] Torres-Boncompte J. Catalá-Gregori P. Garcia-Llorens J. Soriano J. M. Sevilla-Navarro S. (2023). Simultaneous *Salmonella* and bacteriophage isolation on modified semisolid Rappaport Vassiliadis media. Poult. Sci. 10:102960. doi: 10.1016/j.psj.2023.102960 PMC10429287 37579648

[ref71] Trifinopoulos J. Nguyen L. T. von Haeseler A. Minh B. Q. (2016). W-IQ-TREE: a fast online phylogenetic tool for maximum likelihood analysis. Nucleic Acids Res. 44, W232–W235. doi: 10.1093/nar/gkw256 27084950 PMC4987875

[ref72] Vandeplas S. Dubois Dauphin R. Beckers Y. Thonart P. Théwis A. (2010). Salmonella in chicken: current and developing strategies to reduce contamination at farm level. J. Food Prot. 73, 774–785. doi: 10.4315/0362-028X-73.4.774 20377971

[ref73] Vikram A. Woolston J. Sulakvelidze A. (2021). Phage biocontrol applications in food production and processing. Curr. Issues Mol. Biol. 40, 267–302. doi: 10.21775/cimb.040.267 32644048

[ref74] Walker B. J. Abeel T. Shea T. Priest M. Abouelliel A. Sakthikumar S.. (2014). Pilon: an integrated tool for comprehensive microbial variant detection and genome assembly improvement. PLoS One 9:e112963. doi: 10.1371/journal.pone.0112963 25409509 PMC4237348

[ref75] White A. P. Gibson D. L. Kim W. Kay W. W. Surette M. G. (2006). Thin aggregative fimbriae and cellulose enhance long-term survival and persistence of Salmonella. J. Bacteriol. 188, 3219–3227. doi: 10.1128/JB.188.9.3219-3227.2006 16621814 PMC1447457

[ref76] World Health Organization (2018). *Salmonella* (non-typhoidal). Available at: https://www.who.int/news-room/fact-sheets/detail/salmonella-(non-typhoidal)

[ref77] Yukgehnaish K. Rajandas H. Parimannan S. Manickam R. Marimuthu K. Petersen B.. (2022). PhageLeads: rapid assessment of phage therapeutic suitability using an ensemble machine learning approach. Viruses 14:342. doi: 10.3390/v14020342 35215934 PMC8879740

